# Dementia care in gerontological social work: emerging issues and challenges in Saudi Arabia

**DOI:** 10.3389/fpubh.2023.1167856

**Published:** 2023-08-08

**Authors:** Sultan Ali Shubair

**Affiliations:** Social Studies Department, College of Humanities and Social Sciences, King Saud University, Riyadh, Saudi Arabia

**Keywords:** dementia, social work in gerontology, older adults, care challenges, Saudi Arabia

## Abstract

The issues and challenges in the current state of gerontological social work policy, practice, and education related to dementia care in Saudi Arabia are discussed in this article. The following primary issues were explored: (1) the impact of the biomedical model’s global dominance on gerontological social work policy and research for dementia care and health promotion; (2) the position of the older adults in Middle Eastern nations and its connection to the lack of gerontological social work policies, programs, and care services for older adults with dementia and their family caregivers; (3) the effect of the profession of social work’s lack of recognition on the potential evolution of gerontological social work practice in dementia care; (4) the state of dementia patients’ rights, dementia patients’ safety, and dementia patients’ rights to self-determination on the gerontological social work support provided for older adults with dementia; (5) the unequal distribution of dementia care resources and gerontological social work; and (6) the social work education programs’ inability to supply the market with sufficient number of skilled gerontological social workers and its effect on the advancement of dementia care in gerontological social work practice. Approaches for advancing policy, practice, and education are provided to support the evolution of gerontological social work in dementia care in the region.

## 1. Introduction

In Saudi Arabia, there were more than one million and a half individuals aged 60 and over in 2022 (1). By 2050, ten million people are anticipated to be over the age of 60 in the country (2). With the Saudi population’s anticipated move toward old age, chronic diseases, particularly dementia, are anticipated to become more prevalent (3). There are currently no published studies in the nation that evaluate the incidence and prevalence rate of dementia. Dementia is a term that refers to a specific set of symptoms, including memory, language, problem-solving, and other thinking skills impairments. These symptoms are linked to many problems that make life more stressful for older adults with dementia and their family caregivers, such as physical decline, personality change, poor oral intake, depression, anxiety, and sleep disturbance (3–5).

Generally, formal care and services for older adults in the country has enhanced recently *via* introduction of various care and services by government and private sector, such as health services (therapeutic programs, geriatric clinics, and discount cards for medical services), social services (free transportation services, housing programs, and social entertainment programs), educational services (literacy programs, vocational training programs, and teaching Quran reading programs), and spatial services (seating for the older adults, private parking, and wheelchair services) (6). However, more attention and efforts are still needed to address the gaps in diagnosis, health, social, and educational services and care programs specific to older adults with dementia in Saudi Arabia and their informal caregivers (7, 8).

Although there is no official data that show the exact number of informal caregivers for older adults with dementia in the country, Saudi experts predict that most of the care for this population is provided by unpaid informal caregivers due to the cultural and religious belief that caring for older adults is a family responsibility (9). The majority of informal caregivers of older adults with dementia are women, 50 years of age or younger, married, caring for parents, employed, with modest incomes, provide care all the time, and live with the care recipient (10–12).

By offering dementia care, primarily social, psychological, emotional, financial, and spiritual support and care, gerontological social workers, may enhance the quality of life for this population and their family caregivers while also improving the experience of living with dementia. For instance, gerontological social workers might provide this population the proper educational resources to deal with dementia and navigate the challenges associated with it. Gerontological social workers may also encourage older adults with dementia and the people who care for them to join social support groups in order to deal with dementia-related stress. Gerontological social workers may also educate society about dementia in order to lessen the stigma associated with it, which may negatively affect the quality of life for those living with it and their families. To address societal, cultural, and workforce issues associated with dementia care, Gerontological social workers may also promote social policies and programs for this particular population. These duties and responsibilities necessitate a thorough understanding of the disease, expertise in developing interventions that support older adults with dementia and the family caregivers in making emotional adjustments, and familiarity with the available services and practical resources for this population (13, 14).

Although there are many opportunities for gerontological social workers to improve the quality of life for older oldest with dementia and their families, there is still confusion about how to perform gerontological social work with this group, and there is no core set of competencies for such practice in Saudi Arabia. Dementia care and gerontological social work practice and policy for this population are negatively impacted by the global roles within the healthcare system that is informed by the biomedical disease model of dementia, the status of the older adults in Middle Eastern countries, the local lack of recognition of the social work profession, and the lack of gerontological social work education programs (9). The following sections address issues and challenges in gerontological social work policy, practice, and education linked to dementia care. A set of recommendations is also provided to overcome remaining gaps in social work and dementia care.

## 2. Policy issues and challenges related to dementia care in gerontological social work in Saudi Arabia

Middle Eastern cultures, particularly Saudi culture, place high importance on the older adults. They are respected and regarded as a source of blessing, wisdom, and affection. Furthermore, in Middle Eastern culture, taking care of the older adults is a religious and familial obligation. Therefore, it is seen to be a violation of family and religious obligations to send older adults with dementia to nursing homes or long-term care facilities. This viewpoint, which led to a gap in policies, programs, and services for the older adults with dementia and their families, is problematic because it presumes that families can care for their loved ones with dementia without official assistance and support (9). The desire of the family to care for their loved ones with dementia at home should be respected. However, it is also essential to guarantee high-quality care and support for this vulnerable population. Therefore, it is crucial to develop particular gerontological social work policies that offer educational, emotional, social, and financial support and assistance to families caring for older adults with dementia at home.

Because of the belief that focusing on medical research will result in discovering a cure, saving money and patients’ lives, the biomedical model’s popularity around the world has led to an emphasis on helping medical experts and funding their research (4, 7). This assumption needs to be challenged for several reasons. First, it undervalues the high price of developing a dementia cure and the anticipated high cost of the promised treatments (15). Second, because of this assumption, gerontological social work research on non-pharmacological therapies, which is expected to be more successful and cost-effective in improving the quality of life, care, and satisfaction of older adults with dementia and their families until finding a cure, has been underfunded (16). This assumption also fails to consider the challenging living conditions that older adults with dementia and those who care for them currently face, including their emotional, social, and financial challenges. Additionally, it undermined social care policies intended to improve assistance for older adults with dementia and their families by underfunding gerontological social work research programs that explore the psychological and social aspects of dementia (7). It is more appropriate to address dementia and its impacts by concentrating on raising the standard of social care and living for older adults with dementia while working to discover a cure.

The biological trend toward developing a cure to treat dementia resulted in the belief that doing so is the best method to deal with dementia, which encourages medical researchers to request more funding for their work in this field (17). There are several reasons why this assumption has to be challenged. First, it ignores the need for social care research, regulations, and initiatives to advance health, especially brain health, which can help reduce cognitive, functional, and psychological impairments brought on by aging and improve overall body health (18, 19). Second, this assumption ignores the advantages of multidomain lifestyle interventions, including gerontological social work interventions in lowering the risk of cognitive decline in older adults with a high risk of dementia (20, 21). Therefore, it is essential to invest in supporting a healthy lifestyle and brain health for those who are at high risk of dementia while trying to develop a cure to treat dementia.

## 3. Practice issues and challenges related to dementia care in gerontological social work in Saudi Arabia

The number of social work practitioners working in many important social work areas, including gerontological social work area remains deficient due to the need for more recognition of social work as a profession and the scarcity of policies limiting social work jobs to certified social workers. This reality resulted in a shortage of gerontological social workers who offer services and promote care for older adults with dementia and their families (22). The problem is that most employees lack gerontological social work training, which undoubtedly affects dementia services and care across the nation. To fully realize the potential of gerontological social workers in dementia care, initiatives for educating and recruiting more of them in hospitals and social institutions must be promoted.

Gerontological social workers are employed in hospitals to collaborate with multidisciplinary care teams that include medical professionals, formal caregivers, and other non-physician health care providers to extend effective dementia care and services for older adults and family caregivers (23–25). However, gerontological social workers in the region face limitations and barriers that affects their ability to perform their duties appropriately. For instance, they face strong resistance when they try to participate in a patient’s treatment due to the ineffective assumption made by medical physicians that dementia is a medical issue and that social workers’ engagement in dementia cases is unnecessary (26, 27).

In order to ensure that gerontological social workers participate actively and collaborate as a cohesive team in an interdisciplinary healthcare team and achieve the best possible support and care outcomes for older adults with dementia, it is crucial to empower gerontological social workers and raise awareness of hospitals administrations and medical professionals about the significance of the social work profession. This is done by connecting the complex biological components of dementia with the psychological, emotional, social, and cultural factors that contribute to the stressful living experience with dementia.

Disagreement over treatment and care decisions, made by professional physicians among people with dementia, family caregivers, and health care providers, including social workers, is also one of the most critical issues in dementia care (28). The right to self-determination of older adults with dementia, especially those with severe dementia, is threatened by this issue. Additionally, it affects the standard of dementia care and increases moral distress of social workers, especially when; (1) the autonomy of older adults with dementia, such as their decision where to live conflicts with family caregivers, healthcare professionals, and social workers’ desire to keep them safe, (2) the difficulty to assess and understand autonomy of older adults with dementia due to cognitive decline lead to conflict among family caregivers, healthcare professionals, and social workers about what they consider is best for the older adults with dementia’s well-being, and (3) the autonomy of older adults with dementia, such as their decision not to go to a nursing home conflicts with the self-interests of family caregivers (29). This issue, which becomes more complicated with the unclarity of job descriptions (28), needs to be addressed with respect to the right to self-determination for the older adults with dementia.

Despite the fact that social workers have an ethical duty to protect patient’s rights, especially older adults with dementia who are particularly susceptible to physical and medical harms because of their vulnerability (30), the issue of dementia patients’ rights is one of the challenges facing social work providers in the country, especially gerontological social workers. Although the Saudi Patient’s Bill of Rights and Responsibilities included the general rights for older adults patients, (31), the specific rights for dementia patients were not discussed. It is crucial to create and endorse a Dementia Bill of Rights that ensured the rights of older adults with dementia, such as the rights to be informed of their diagnosis, to receive quality medical care and treatment, and to be provided with information and support they need to participate as fully as possible in care decisions in all stages of dementia.

Another concern that hospital social workers and medical personnel must address is patient safety (32). Some of the hospitals in the country place a low priority on patient safety, which includes providing adequate assessment, treatment, and intervention, reporting error events, responding to errors, being open with patients, ensuring impartiality, and providing adequate management support for patient safety (33). Patient’s psychological, emotional, and social well-being is in risk as a result of the violations of patient safety-related issues, especially older people with dementia (32). Promoting and advocating for policies to reinforce the patient safety culture in hospitals is imperative to alleviate concerns and enhance the quality and safety of care for older adults with dementia and their family caregivers.

Access to resources is also one of the critical issues related to dementia care in Saudi Arabia. For instance, the majority of medical and healthcare resources are located in urban cities, which makes it difficult for gerontological social workers to provide those living in rural areas with appropriate resources to deal with dementia challenges (32). For citizens and residents to have access to high-quality medical care and services, there must be an equitable allocation of resources across the nation.

## 4. Education issues and challenges related to dementia care in gerontological social work in Saudi Arabia

Due to several reasons, social work education programs could not adequately equip their students to meet this demand and need of gerontological social workers who are specialized in providing care and services to older adults with chronic diseases, such as dementia. First, few faculty members in the country specialize in geriatric social work. Furthermore, social work education programs in the universities do not offer foundational courses to train students who want to focus on serving and caring for senior populations, particularly those with chronic illnesses like dementia. Furthermore, there are not enough graduate students specializing in gerontology and dementia care research in Saudi Arabia because there are not enough academic mentors in the field (34). To fill the urgent market demand for gerontological social workers and researchers, it is crucial to hire faculty members from other countries with expertise in geriatric social work while also intending to increase the number of faculty members with gerontological social work specialization in Saudi Arabia. Along with that, social work education programs at universities need to create fundamental courses that educate future social workers about aging and chronic illnesses like dementia and its related effects.

The absence of social work scientific literature and knowledge that discuss social difficulties linked with chronic diseases, such as dementia, is also one of the challenges that social work education in Saudi Arabia faces (17, 35). This fact heightens the propensity of academics to use outdated materials and information that are not culturally relevant, which has an impact on student learning outcomes and the establishment of a gerontological social work education foundation in the country that fits its unique culture and addresses the escalating social issues related to aging and chronic diseases like dementia (36). It is significant to develop a foundation for culturally appropriate gerontological social work education and increase the quantity of high-quality social work geriatric research in Saudi Arabia that tackles the most prevalent geriatric health disorders with psychological and social effects, such as dementia.

The limited options for social work students to receive dementia care training in health and social institutions is also one of the issues facing social work education in the country. There are various reasons for this. First, few public and private health/social institutions are eager to offer social work students internship opportunities due to the unfounded concern that they will interfere with these organizations’ principal missions and expose ethical misbehavior and negligence. The lack of field practicum programs in social work education programs that outline student and agency obligations, offer high-quality supervision, and establish a connection between theoretical knowledge, field practicum, and market needs complicates the situation (34). Development of field practicum programs in the social work education programs in Saudi Arabia that gives high priority to field practicum as part of the academic curriculum and addresses the issues related to field practicum education is significant to increase the number of trained and highly skilled gerontological social workers, including those who are specialized in providing care for older adults with dementia and their family caregivers.

## 5. Theoretical perspectives of dementia care and their impacts on dementia care and gerontological social work

The development of dementia-related practice and policy is influenced by how society views and understands dementia. Therefore, changing social work policy, practice, and education related to dementia requires changing the dominant way of understanding it. There are several theoretical models to understand dementia. Each model uniquely affects dementia-specific care practices, policies, and research related to discipline of social work and other disciplines. Theoretical models of understanding dementia can be categorized into four conceptual models: biological model, socio-psychological model, disability model, and social gerontological model (37, 38).

According to the biological approach, dementia is diagnosed as a pathological disorder with treatable symptoms. Thus, in accordance with this model of understanding, dementia is a condition that results in a steady degeneration of the brain. Consequently, medical control is the best strategy for dealing with dementia. Therefore, it is crucial to recognize the signs of this disease in order to ascertain its underlying causes. The development of dementia treatments or preventative measures should be the main focus of all research. Healthcare providers should concentrate on reducing dementia symptoms. The goal of the policymakers should be to create laws that will make it possible to cure dementia. However, the biological approach has been criticized for prioritizing dementia research over offering patients and their families the assistance and care required to deal with the illness. Social-psychological and disability models, which prioritize enhancing the health and quality of life of people with dementia, were developed in response to this critique of the biology paradigm (37, 38).

The social-psychological model focuses on how dementia affects both the individuals with dementia and the family members who provide care for them. Because of this, this paradigm perceives dementia as an individual-level experience that affects people differently. This perspective view assisted people living with dementia and their carers manage the condition and its consequences. According to this paradigm, the research aims to develop treatments and preventative measures for dementia patients. It is the duty of healthcare providers, including social workers and caretakers, to work with older adults with dementia to provide activities that take into account their preferences and needs. By taking into account the unique experiences of older adults with dementia and family caregivers, the social psychological approach focuses on creating social policies intended to promote medical and non-medical interventions (37, 38).

According to the disability paradigm, dementia is a disorder that causes a disability. However, it does not consider disability as a problem. Instead, by neglecting to change social attitudes and the environment for dementia patients, society has problematized impairment and excluded older adults with dementia from fully participating in society. In order to properly stimulate and engage older adults with dementia in social situations, environmental adjustments are needed. As a result, the fundamental objective of the research, treatment strategies, and legislation should be to maintain the independence of older adults with dementia for as long as feasible by promoting non-medical interventions (37, 38).

The social gerontological model indicates that life with dementia can remain fulfilling if society does not exclude and isolate older adults with dementia and perceive them as a burden. Thus, societies’ social, environmental, and structural contexts influence the living experience with dementia. Therefore, dementia research, healthcare practices, and policies should focus on understanding the broader social and structural factors that impact the living experience with dementia, thus promoting positive images of aging to decrease their stressful experience with the disease (37, 38).

The biological paradigm of understanding dementia continues to predominate in the region, despite the fact that other nations, including the United States, have begun to push an integrative model to understand dementia (37). The supremacy of the biological model has an impact on social services, policies, and social work practice with older adults with dementia and their family caregivers. It undermined the social services and assistance provided to older adults living with dementia to lessen the emotional, social, psychological, and economic strain of the disease. It also led to the lack of national guidelines, educational programs, and social service initiatives for older adults with dementia and their family caregivers. It also contributed to the lack of financing for dementia care research and long-term care facilities or nursing homes for older adults with dementia. It also expanded the gap between gerontologists and healthcare professionals who focus on providing care for the older adults, such as gerontological social workers and other healthcare providers (4, 5, 7, 11). The following recommendations should be implemented in order to overcome the gerontological social work policy, practice, and education gaps related to dementia care:

## 6. Approaches and recommendations

Integrative biological, psychological, disability, and gerontological models should be used to understand dementia as a condition. To foster a deeper understanding of the nature of living with dementia, social work policy, education, practice, and policy efforts should be concentrated on macro and micro levels. Gerontological social workers should take neurological impairment and social structural limitations into account while providing older adults with dementia and their family caregivers with high-quality care. Social work policymakers should incorporate biomedical information as well as behavioral, social, and structural issues when drafting policies for senior citizens with dementia and the family caregivers who support them.

The country needs to prepare for the increased financial, social, and health burden that will come with the rise in dementia cases. The Ministries of Health and Human Resource and Social Development, social service organizations, medical and non-medical professionals, and researchers should collaborate to assess the current level of all aspects of care for people with dementia and their families and to formulate recommendations for addressing the health, societal, economic, and workforce issues related to dementia. The prior recommendation should be incorporated into the Saudi Vision 2030 by working with the Council of Economic and Development Affairs. Saudi Arabia must also develop and maintain a national plan to deal with dementia, with the explicit goals of identifying long-term care assistance and service gaps, enhancing the standard of social care and quality of life for older adults with dementia and their families, and enhancing social workers’ roles in dementia care.

To meet the needs of older adults with dementia and family caregivers, it is essential to provide social workers with well-designed gerontological social work education programs. In order to provide patient-centered care to people with dementia and their family caregivers, Saudi Arabia should prepare to increase the number of healthcare service providers, including social workers, and establish geriatrics-specific training programs. In order to provide appropriate dementia care and services, Saudi hospitals must establish multidisciplinary care teams that include social workers and other medical professionals. Through promoting a positive image of the older adults with dementia and the caregivers who support them, social work education programs should raise public acceptance and enhance integration. Additionally, it is crucial to use social media to reach a large audience and to take advantage of other media platforms to raise awareness of dementia related concerns and the roles of social workers. Creating dementia-related content and incorporating information on aging, its societal impacts, and the roles of social work in dementia care into university and school curricula is significant.

## Author contributions

The author confirms being the sole contributor of this work and has approved it for publication.

## Conflict of interest

The author declares that the research was conducted in the absence of any commercial or financial relationships that could be construed as a potential conflict of interest.

## Publisher’s note

All claims expressed in this article are solely those of the authors and do not necessarily represent those of their affiliated organizations, or those of the publisher, the editors and the reviewers. Any product that may be evaluated in this article, or claim that may be made by its manufacturer, is not guaranteed or endorsed by the publisher.
